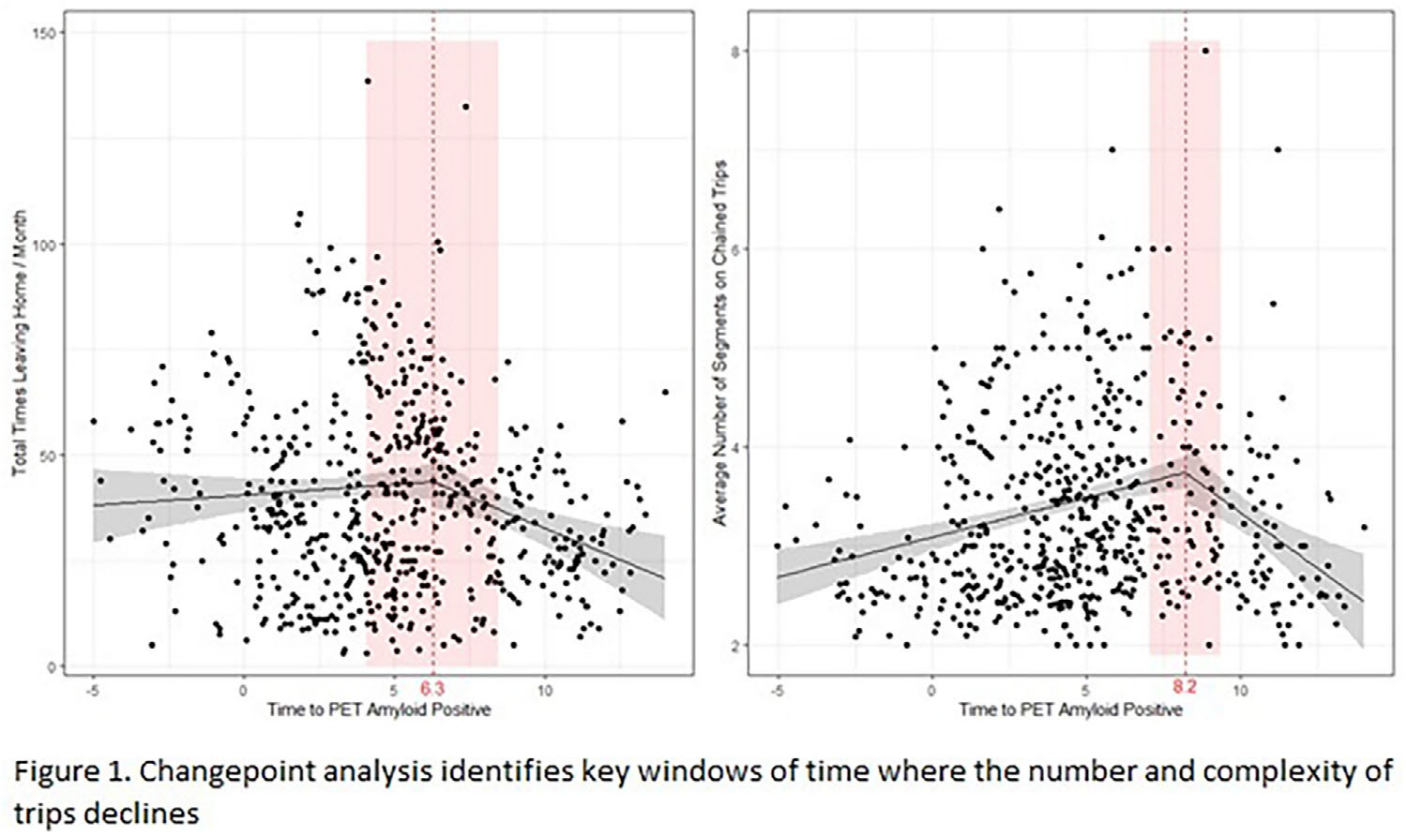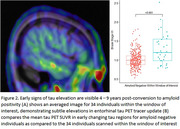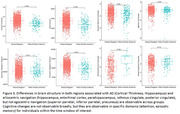# Driving Behavior as a Functional Biomarker of Preclinical Alzheimer Disease

**DOI:** 10.1002/alz.091540

**Published:** 2025-01-03

**Authors:** Julie K. Wisch, John C. Morris, Tammie L.S. Benzinger, Beau Ances, Ganesh M. Babulal

**Affiliations:** ^1^ Washington University in St. Louis School of Medicine, St. Louis, MO USA; ^2^ Knight Alzheimer Disease Research Center, St. Louis, MO USA; ^3^ Washington University School of Medicine, Saint Louis, MO USA; ^4^ Washington University School of Medicine, St. Louis, MO USA

## Abstract

**Background:**

Trip chaining occurs when a driver departs from an origin and travels to multiple locations before returning. Increased trip complexity may require higher levels of executive function, memory, and navigational abilities. Subtle behavioral changes are apparent before a clinical diagnosis of Alzheimer Disease (AD); however, the correspondence between preclinical AD pathology (amyloid deposition, tauopathy, neurodegeneration), cognition, and changes in trip chaining behavior is unknown.

**Method:**

46 individuals enrolled in the DRIVES study (µ_Enrollment_ = 2.7 years, ơ_Enrollment_ = 1.1 years) at Washington University in St. Louis completed longitudinal amyloid PET imaging and converted from amyloid negative (A‐) to amyloid positive (A+). All data were ordered temporally relative to date of conversion. We fit changepoint models to the average number of segments per departure and average number of trips away from home per month over time. After identifying key windows‐of‐change in trip‐related behavior, we extended our analysis of neuroimaging and cognitive data to all Knight Alzheimer Disease Research Center participants. We used a published analytical model to estimate time from A+ for all participants with amyloid PET (N = 1082). Then, we evaluated the spatial deposition of tau for individuals imaged within the time window‐of‐interest identified via changepoint model (N = 34). We applied linear mixed effects models to compare measures of brain structure and cognition between A‐ controls (N = 752, 195 respectively) and individuals within the window‐of‐interest (N = 105, 66).

**Result:**

The total number of departures declined 6.3 (95% CI: 4.2, 6.7) years after becoming A+, and the number of segments per trip declined 8.2 (95% CI: 7.0, 9.3) years after becoming A+ (Figure 1). Within this key window (4.2 – 9.3 years post‐A+), tau was elevated in the earliest anticipated regions (Braak I/II), and 24/34 individuals were tau positive (Figure 2). Neurodegeneration was observable in the cortex, hippocampus, and regions associated with allocentric navigation but not egocentric navigation. Cognitive changes are not seen globally but were seen in attention and episodic memory (Figure 3).

**Conclusion:**

Changes in amyloid burden, tauopathy, and neurodegeneration have real‐world effects on daily driving behavior even before global cognitive impairment is observed.